# Regional hotspots for chronic kidney disease: A multinational study from the ISN-GKHA

**DOI:** 10.1371/journal.pgph.0004014

**Published:** 2024-12-05

**Authors:** Pablo Garcia, Anna K. Strasma, Eranga Wijewickrama, Silvia Arruebo, Fergus J. Caskey, Sandrine Damster, Jo-Ann Donner, Vivekanand Jha, Adeera Levin, Masaomi Nangaku, Syed Saad, Marcello Tonelli, Feng Ye, Ikechi G. Okpechi, Aminu K. Bello, David W. Johnson, Shuchi Anand

**Affiliations:** 1 Division of Nephrology, Department of Medicine, The University of New Mexico School of Medicine, Albuquerque, New Mexico, United States of America; 2 Division of Nephrology, Department of Medicine, Duke University School of Medicine, Durham, North Carolina, United States of America; 3 Duke Global Health Institute, Durham, North Carolina, United States of America; 4 Department of Clinical Medicine, Faculty of Medicine, University of Colombo, Colombo, Sri Lanka; 5 University Medical Unit, National Hospital of Sri Lanka, Colombo, Sri Lanka; 6 National Institute of Nephrology, Dialysis & Transplantation, Colombo, Sri Lanka; 7 The International Society of Nephrology, Brussels, Belgium; 8 Population Health Sciences, Bristol Medical School, University of Bristol, Bristol, United Kingdom; 9 George Institute for Global Health, University of New South Wales (UNSW), New Delhi, India; 10 School of Public Health, Imperial College, London, United Kingdom; 11 Manipal Academy of Higher Education, Manipal, India; 12 Division of Nephrology, Department of Medicine, University of British Columbia, Vancouver, British Columbia, Canada; 13 Division of Nephrology and Endocrinology, The University of Tokyo Graduate School of Medicine, Tokyo, Japan; 14 Division of Nephrology and Immunology, Faculty of Medicine and Dentistry, University of Alberta, Edmonton, Alberta, Canada; 15 Department of Medicine, University of Calgary, Calgary, Alberta, Canada; 16 Canada and Pan-American Health Organization/World Health Organization’s Collaborating Centre in Prevention and Control of Chronic Kidney Disease, University of Calgary, Calgary, Alberta, Canada; 17 Division of Nephrology and Hypertension, University of Cape Town, Cape Town, South Africa; 18 Kidney and Hypertension Research Unit, University of Cape Town, Cape Town, South Africa; 19 Department of Kidney and Transplant Services, Princess Alexandra Hospital, Brisbane, Queensland, Australia; 20 Centre for Kidney Disease Research, University of Queensland at Princess Alexandra Hospital, Brisbane, Queensland, Australia; 21 Translational Research Institute, Brisbane, Queensland, Australia; 22 Australasian Kidney Trials Network at the University of Queensland, Brisbane, Queensland, Australia; 23 Division of Nephrology, Department of Medicine, Stanford University School of Medicine, Stanford, California, United States of America; PLOS: Public Library of Science, UNITED STATES OF AMERICA

## Abstract

Chronic kidney disease (CKD) disproportionately affects certain populations as demonstrated by well-established subnational geographic hotspots of CKD in Central America and South Asia. Using data from the third iteration of the International Society of Nephrology Global Kidney Health Atlas (ISN-GKHA), we aimed to systematically identify sub-national geographic or population clusters with high prevalence of CKD. The ISN-GKHA survey was conducted from July to September 2022, and included questions regarding whether a regional CKD hotspot existed in the respondents’ country and possible contributors. A CKD hotspot was defined as a population cluster with a high risk of kidney failure requiring dialysis or transplant, or people dying from kidney failure. Overall, 46 out of 162 responding countries reported subnational hotspots for CKD within their country. Hotspots were reported across all regions, except for the Middle East. Latin America had the highest percentage (12 of 21, 57%) of countries reporting a regional CKD hotspot followed by the regions of North and East Asia, and Western Europe. Adults aged 18 to 44 years and rural populations were most commonly identified as the primary groups affected. Clinical factors were most commonly identified as contributors to CKD (hypertension in 74% and diabetes in 72%), followed by cultural (e.g., diet and herbal medications in 67%), and environmental (e.g., polluted water in 43%) factors. Latin American countries more commonly reported climate, cultural, and environmental factors as contributors compared to other regions. Across the world, there are a number of subnational regions where in-country experts identify a disproportionately higher burden of CKD, commonly occurring among younger age groups with clinical, cultural, and environmental contributors specific to these geographic regions. In-depth studies, starting with systematic epidemiology efforts, are needed to investigate the aetiopathogenesis of these CKD hotspots around the world so that tailored interventions can be offered.

## Introduction

Chronic kidney disease (CKD) prevalence has increased over the past two decades [1, 2]. Furthermore, CKD has emerged as one of the leading causes of death in the world, ranking as the 12th leading cause of death in 2017 [1]. Although country-wide estimates of CKD prevalence report that around 10% of the global population is affected [3], within-country heterogeneity in CKD prevalence, cause, and mortality likely exists. For example, in Central America and South Latin America, CKD ranks as the second and fifth in the list of causes of death, respectively [1]. In some countries within Latin America, a kidney disease not associated with diabetes, hypertension or glomerular disease, termed Mesoamerican Nephropathy (MeN), exists at high prevalence within coastal regions [4–7]. A similar profile of kidney disease exists in other regions including Uddanam Nephropathy in Central India [8] and Sri Lankan nephropathy in Sri Lanka [9]. These regions have been broadly classified as hotspots of chronic kidney disease of unknown etiology (CKDu). Risk factors CKDu include an occupation involving strenuous labor in high temperatures, applying agrochemical pesticides without protection, and drinking untreated water [4–6, 10, 11].

CKDu was recognized in the early twenty-first century due to astute observations from clinicians working in affected areas. Whether similar phenomena of subnational hotspots of CKD exist elsewhere is uncertain, partly due to limited capacities of CKD detection, and cause of death attribution [12–16]. Furthermore, CKD detection is not often included in large-scale epidemiological studies, even in countries implementing large-scale diabetes or cardiovascular risk assessment surveys [17]. With the hypothesis that CKDu exemplifies a potentially broader phenomenon of subnational variation in kidney disease prevalence and mortality, we evaluated data from the third iteration of the International Society of Nephrology Global Kidney Health Atlas (ISN-GKHA) to describe hotspots for CKD across the world. This analysis, based on survey data from local kidney disease stakeholders, may provide the earliest signal to the global nephrology community on the locations and populations disproportionately affected by CKD. The ISN-GKHA captures insights from diverse regions, especially those where academic research, surveillance systems, and electronic health records are limited. Additionally, assessing the local stakeholders’ perspective on the underlying etiologies of these hotspots provides insight into possible preventative efforts and research collaborations.

## Methods

### Approach

The ISN-GKHA is a cross-sectional survey conducted by the ISN. Two previous iterations using the same standardized methodology have been published [18, 19]. Detailed methods for this third iteration are described elsewhere [3]. Briefly, the current study relied on a multinational survey of nephrology specialists with knowledge of the status and extent of national kidney care practices from each country. Key stakeholders in kidney care were identified from each country to participate, including a nephrology professional society leader, a policymaker, and a leader of a national or regional patient representative organisation. In some instances, country stakeholders had multiple roles (e.g., both nephrology leader and policymaker). The survey was coordinated through the ISN’s 10 regional boards: Africa, Eastern and Central Europe, Latin America, the Middle East, Newly Independent States (NIS) and Russia, North America and the Caribbean, North and East Asia, Oceania and South East Asia (OSEA), South Asia, and Western Europe.The survey was conducted from June 1 to September 30, 2022. During this period, intensive follow-ups were conducted by email and telephone with ISN regional and national leaders to ensure complete and timely responses.

### Survey instrument development and validation

The current survey was developed, and peer reviewed, by the ISN executive committee, regional leaders, and international collaborators to ensure comprehensiveness before being reviewed by the 10 ISN regional boards. The English language survey was translated into French and Spanish. The component focused on identifying potential subnational hotspots is available at https://www.theisn.org/initiatives/global-kidney-health-atlas/. A set of questions in the survey focused on within-country regional kidney disease hotspots, defined as population clusters with high risk of kidney failure requiring dialysis or transplant, or people dying of kidney failure. The survey included questions with both multiple-choice and free-text responses about the specific location, the age groups affected, likely contributors to the disease process, types of industries found in the regions affected, and the predominant climate and altitude found in the country. Multiple selections were permitted for the questions pertaining to age groups, causes, and industries associated with CKD hotspots.

### Statistical analysis

Using country as the unit of analysis, responses were summarized based on the key survey domains using a descriptive statistical approach and reported as counts with percentages. Results were stratified by ISN region and by World Bank income group (estimated in June 2022). Free text responses were summarized through description and graphing. The results were examined based on a pre-existing protocol and reported according to the Guidelines for Accurate and Transparent Health Estimates Reporting (GATHER) statement [20]. The analysis was conducted using STATA 17 software (Stata Corporation, 2017) and Microsoft Excel.

### Ethics approval

The University of Alberta Research Ethics Committee approved this project (protocol number: PRO00063121). Consent was not required by survey respondents. Our study did not report experiments on humans and/or the use of human tissue samples.

## Results

### Country and ISN regional distribution

**Fig 1:**
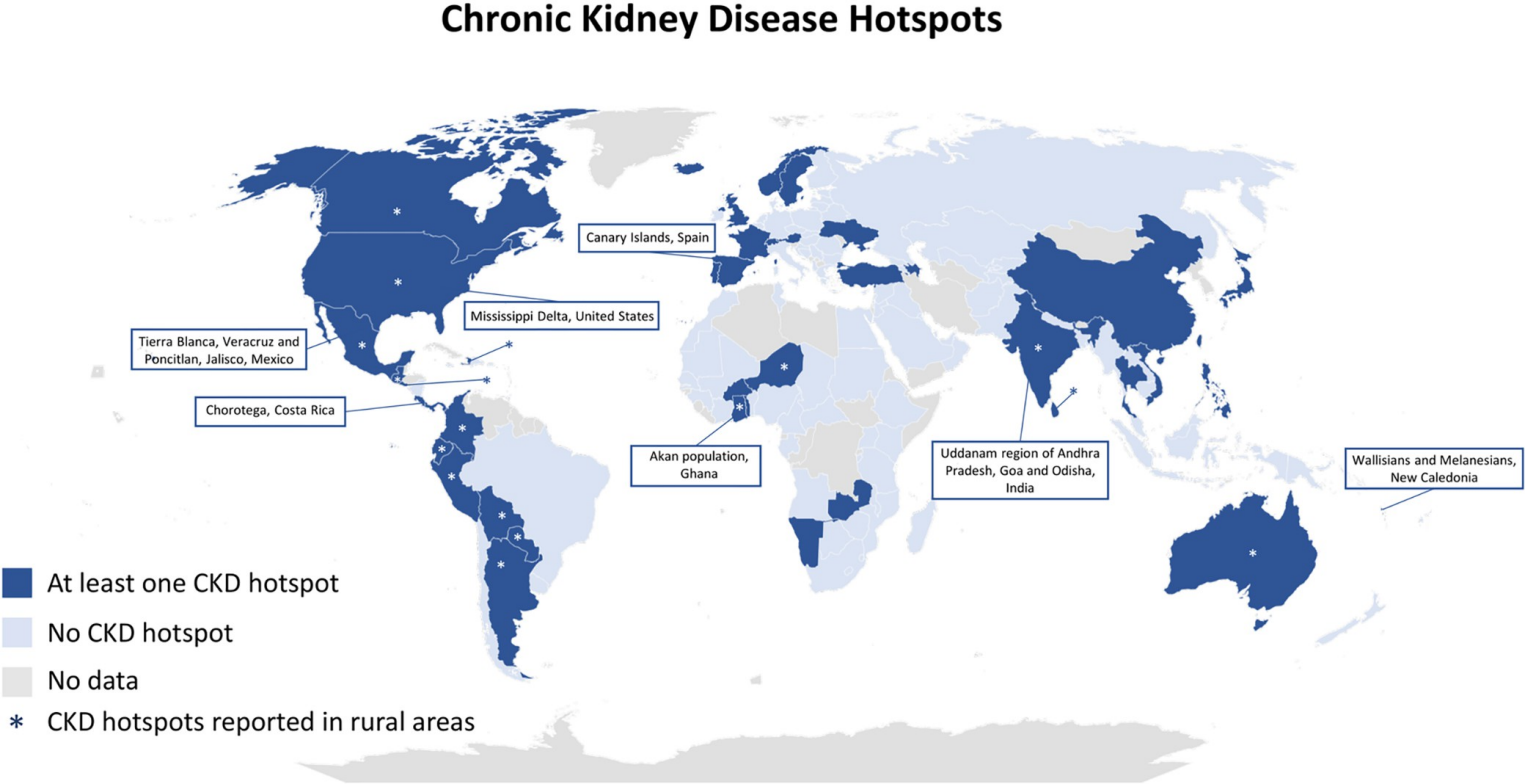
Chronic kidney disease hotspots. Abbreviation: CKD = chronic kidney disease. *Chorotega region is located within the Guanacaste province. This map was created using Microsoft Excel powered by Bing. (https://www.microsoft.com/en-us/maps/bing-maps/product#:~:text=(b)%20Attribution%20and%20proprietary%20notices,party%20attribution%20provided%20by%20Microsoft).

Out of 167 countries included in the ISN-GKHA, 162 (97%) responded to questions related to subnational hotspots of kidney disease. Forty-six (28%) participating countries reported a regional variation in rates of kidney disease within their countries (Table 1, Fig 1). There was complete internal consistency for this question among respondents from the same country regardless of role. Latin America had the highest percentage (12 of 21, 57%) of countries reporting a regional CKD hotspot followed by the regions of North and East Asia, and Western Europe. The countries reporting CKD hotspots were distributed across every country income level with the highest proportion (14 of 37, 38%) from upper-middle income countries (Table 1). CKD hotspots were most often reported in countries where the leading causes of kidney failure were diabetes or hypertension.

**Table 1:** Presence of regional hotspots of CKD by ISN region and World Bank income groups (N, %).

	Regional Variation (n (%))				Total Countries
	No		Yes		
	N	%	N	%	
**Overall**	**116**	**(72)**	**46**	**(28)**	**162**
**ISN regions:**					
Africa	33	(85)	6	(15)	39
Eastern and Central Europe	15	(94)	1	(6)	16
Latin America	9	(43)	12	(57)	21
Middle East	11	(100)	0	(0)	11
NIS and Russia	8	(80)	2	(20)	10
North America and the Caribbean	8	(67)	4	(33)	12
North and East Asia	3	(50)	3	(50)	6
Oceania and South East Asia	12	(67)	6	(33)	18
South Asia	5	(71)	2	(29)	7
Western Europe	12	(55)	10	(45)	22
**World Bank income groups:**					
Low income	14	(78)	4	(22)	18
Lower-middle income	34	(77)	10	(23)	44
Upper-middle income	23	(62)	14	(38)	37
High income	45	(71)	18	(29)	63

Abbreviations: CKD–chronic kidney disease; ISN–International Society of Nephrology; NIS–Newly Independent States

### CKD hotspots geographic regions and populations

Specific geographic areas, resident zones (i.e. rural vs. urban), and sociodemographic populations were reported to suffer from disproportionate burden of CKD based on free-text survey responses (Fig 1, S1 Table). Specific geographic areas included previously-established CKDu hotspots, such as the Chorotega region which is located within the Guanacaste province (Costa Rica), Tierra Blanca (Mexico), Uddanam, Goa, and Odisha (India), and the southern coast of Guatemala. Other geographic regions reported as CKD hotspots included the Canary Islands (Spain), Grenadine Island (St. Vincent and the Grenadines), North Peru, Center and South Panama, sea-level Ecuador, the Pacific coast of Colombia, North Togo, Mississippi delta (USA), South Taiwan, North and Northeast Thailand, and the hot and dry regions of Japan. Notably, there were no CKD hotspots identified in Nicaragua.

Rural areas were commonly reported as the sites of CKD hotspots (16 countries, Fig 1), while urban areas were reported as the sites of CKD hotspots in Namibia, Zambia, and Norway (S1 Table). Populations with reported disproportionate burden of CKD included Black or South Asian populations in the United Kingdom, clusters of families with polycystic and interstitial kidney disease in Iceland, First Nations populations in Canada, Aboriginal and Black populations in Colombia, the Akan ethnic population in Ghana, and the Wallisian people in New Caledonia. Respondents also reported disproportionate burdens of CKD in populations living in socioeconomically deprived areas in the following 9 countries: Argentina, Australia, Ecuador, Haiti, India, Portugal, Sweden, Ukraine, and the United States.

### Age distribution

**Fig 2:**
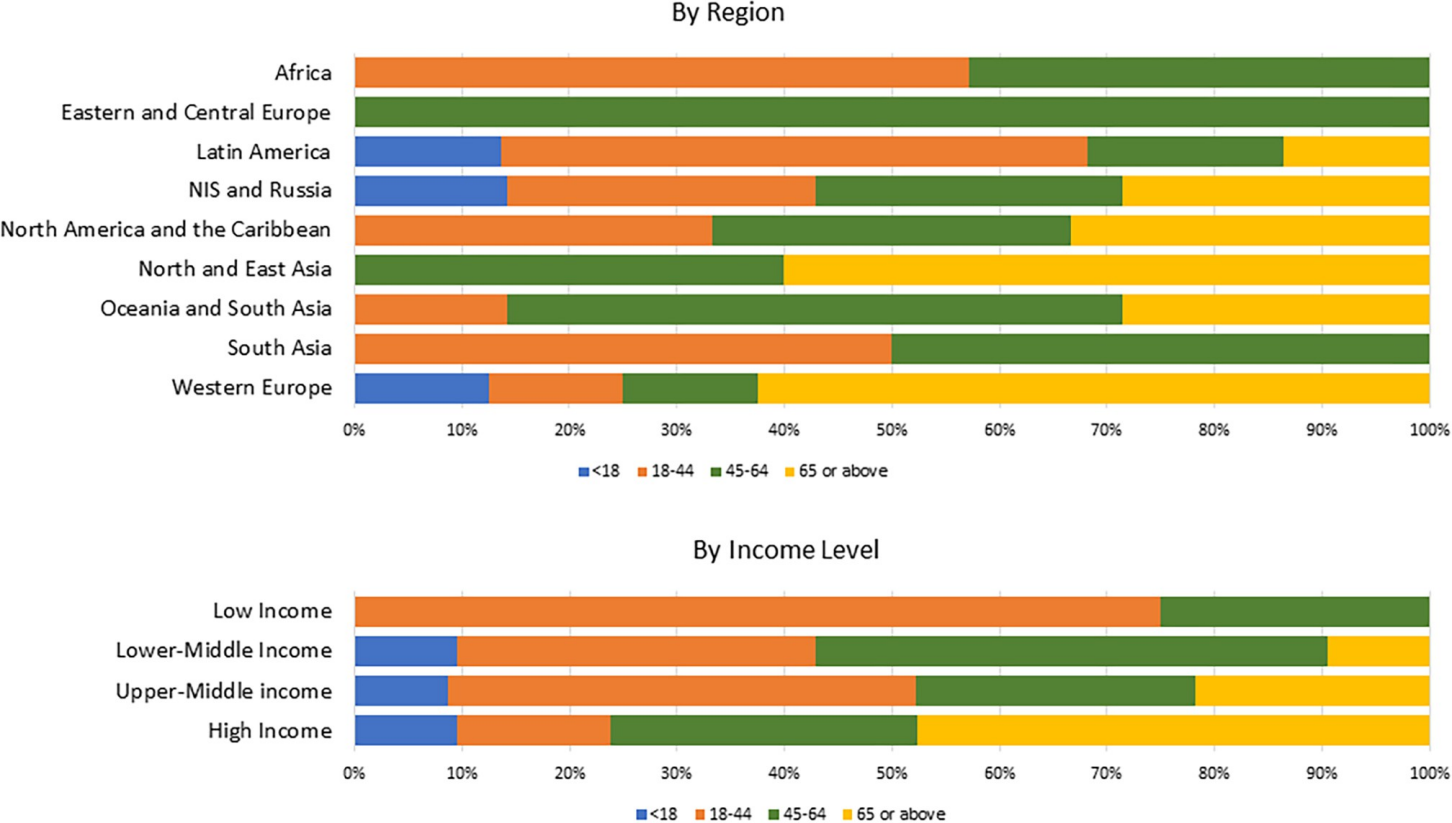
Age categories affected by CKD categorized by (A) ISN regions and (B) by World Bank income groups. Abbreviations: CKD = chronic kidney disease; ISN = International Society of Nephrology; NIS = Newly Independent States.

Regional CKD hotspots were most commonly reported to affect 18–44-year-olds (Fig 2). In two regions—North and East Asia, and Western Europe—CKD hotspots were reported to occur mostly in the 65+ years age group (Fig 2).

### Contributors to CKD

**Fig 3:**
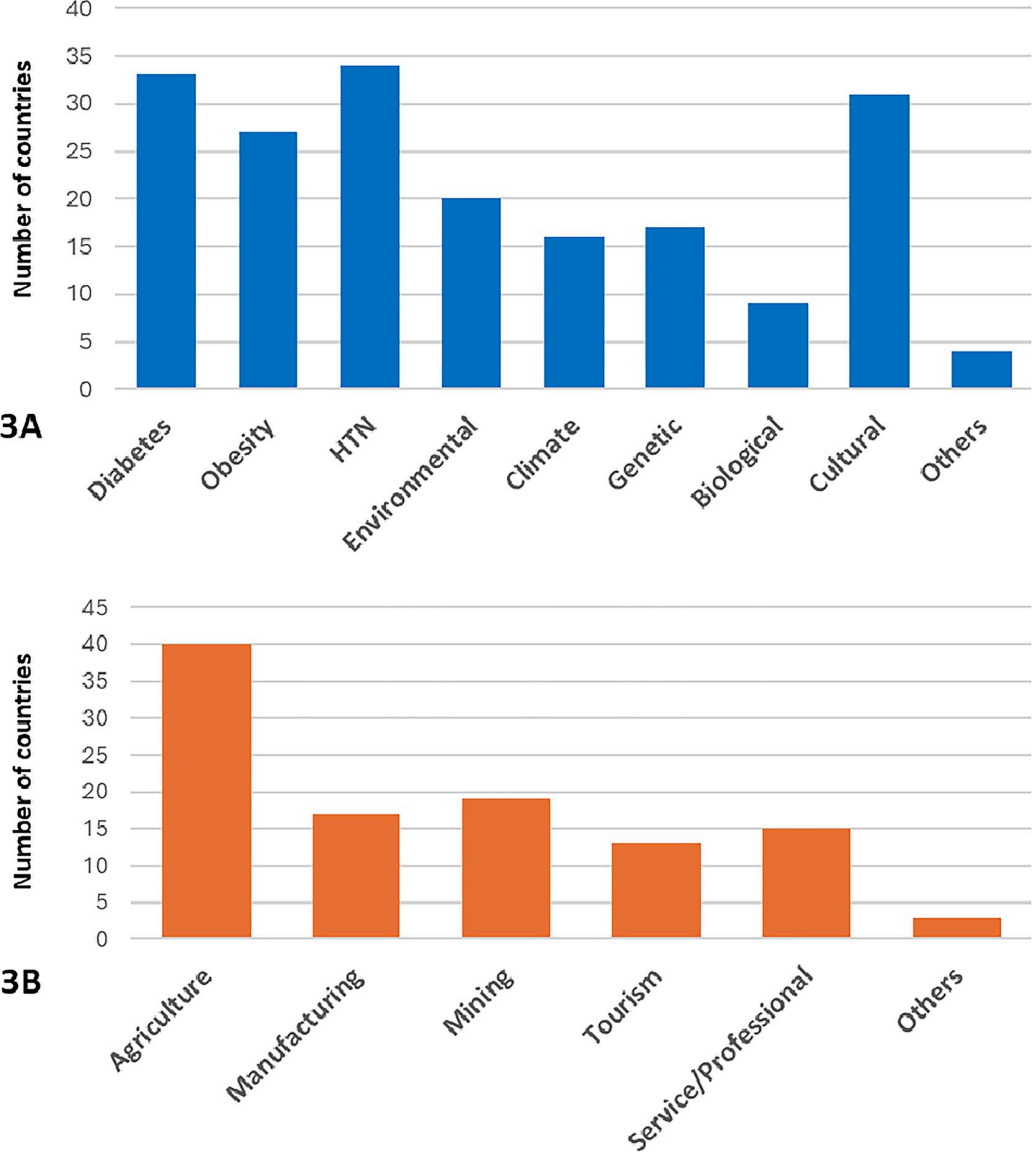
3A and 3B: (3A) Causes of CKD Hotspots and (3B) Industries in CKD Hotspot Areas. Abbreviation: CKD = chronic kidney disease; HTN = hypertension.

The most common reported contributors to CKD hotspots overall were hypertension (34 of 46 countries reporting hotspots, 74%) and diabetes (33 of 46 countries, 72%) (Fig 3A). Notably, after these clinical risk factors, cultural factors, such as diet, use of non-steroidal agents, and herbal medications, were reported to be the most common contributor (31 of 46 countries, 67%). Obesity (27 of 46, 59%), environmental (20 of 46, 43%), and genetics (17 of 46, 37%) were also commonly reported as contributors. Climate (13 of 46, 28%), biological contributors (HIV, tuberculosis, or kidney stones) (9 of 46, 20%), and other causes (4 of 46, 9%) were less commonly reported.

The reported causes of these CKD hotspots differed based on ISN region (S1 Fig), with Latin American region countries more often citing climate and environmental factors. Although diabetes and hypertension were the most commonly cited causes in other regions, 12 countries outside of Latin America reported environment as a contributing factor. The contribution of cultural risk factors was similar between Latin America and other regions.

### Regional industry and climate

Agriculture was the most commonly reported industry among CKD hotspots overall (40 of 46 countries with hotspots, 87%) and for each region. Other industries were much less commonly cited as being associated with CKD hotspots, with the next most frequent industry being mining (19 of 46 countries, 41%) (Fig 3B). CKD hotspots in workers or communities near certain industries were also mentioned in the free-text responses: gold-panning (Burkina Faso), mining (Colombia, Peru), and agriculture (Ghana, Bolivia, Colombia, El Salvador, Guatemala, Paraguay, India, Sri Lanka). Over half the countries with CKD hotspots had a tropical climate (59%), and the most common altitude was sea level (52%). Countries with these CKD hotspots had a mean average annual temperature of 18.3°C, which was cooler than the mean average annual temperature of countries without CKD hotspots (19.7°C) [21].

## Discussion

In this multinational survey of kidney disease in 162 countries, we found that regional hotspots of CKD were common and included countries in most regions and across all income levels. Latin America had the highest proportion of countries reporting regional CKD hotspots. The age group most affected by CKD hotspots was reported to be 18- to 44-year-olds, and clinical factors were the most common contributor to the disproportionate burden of CKD among subnational geographic areas or specific racial or ethnic populations, followed by cultural and environmental factors. Agricultural work was the most commonly associated occupation group. Investigating regional variation in CKD may identify geographic, environmental, and cultural factors that drive CKD incidence and progression, which provides new pathways to mitigate kidney disease-related morbidity and mortality.

The vast majority of data underpinning the current practices in managing kidney disease are derived from North America and Western Europe. Even the measures of kidney function, and the definition of kidney disease versus kidney health, are derived primarily from Caucasian populations. Based on data from these best-studied regions, providers most commonly ascribe kidney disease to either hypertension or diabetes. Existing cohorts of kidney disease generally lack kidney biopsy data, detailed exposure assessments for cultural practices, or occupational or environmental histories, which could potentially accelerate CKD in the background of hypertension or diabetes [22]. Our survey spotlights that these ‘non-traditional’ factors may contribute to disproportionate burdens of kidney disease. Given that some endemic nephropathies, such as Balkan endemic [13, 23] nephropathy and Itai-Itai disease [24], were linked to medication and environmental factors, and therefore amenable to preventive interventions, it is critical to focus on these under-investigated exposures to identify novel, low-cost preventive interventions.

In addition to non-traditional factors that contribute to CKD hotspots, these results highlight that there are disproportionate burdens of traditional CKD within countries. This finding could be due to relatively high prevalences of hypertension, diabetes, cardiovascular disease, and other traditional risk factors in certain areas due to socioeconomic determinants of health, genetic predisposition, or biological factors. The globally increasing prevalence of these CKD-antecedent noncommunicable diseases may be affecting certain vulnerable populations more than others [25, 26]. In addition, the traditional risk factors of diabetes or hypertension may have a higher proclivity for kidney disease manifestation in specific regions or populations within a country. Studying high-risk populations has yielded one of the most important advances in kidney disease—the identification of APOL1 high risk genotypes, for which promising therapies are now under development [27, 28]. Similarly, Hoy et al. focused on the high incidence of kidney failure among the Australian Aboriginal population and delineated the possible contribution of low nephron mass [29]. With access to novel diagnostic approaches, advances in biochemical tissue analysis have identified leukocyte chemotactic factor 2 (LECT2) as a novel subtype of amyloid causing kidney disease, primarily among Hispanic persons in the Southwest region of the United States [30–32]. The survey responses presented here suggest there is an increasing burden of CKD both from the rise of traditional CKD risk factors and from nontraditional factors such as culture and the environment. Both disease pathways should be investigated to implement disease prevention, and to define the etiological factors causing CKD in these populations. Increased CKD surveillance through increased screening (possibly with innovative devices or instruments), registries, and reporting systems is vital to identify and address emerging CKD hotspots.

Another notable finding in our study is the identification of rural areas and poorer populations as subnational CKD hotspots by our survey respondents. One framework for kidney disease casts the disease as a “downstream” or “end-organ” manifestation of cardiovascular disease or diabetes. Global public health surveillance efforts have in part coalesced to a greater degree on these antecedent diseases [33]. Indeed, rural populations appear to be at risk for the development of traditional CKD-antecedent diseases, such as diabetes and hypertension, as unhealthy lifestyle changes (such as increased access to unhealthy, processed food) occur where there is often limited access to medical care. Furthermore, a lack of public infrastructure, medical care, and health literacy may leave them more vulnerable to risk factors such as untreated drinking water, agrochemical exposure, and herbal medication use. Quantifying the association of these risk factors with kidney disease requires not only prioritization by policy makers, but potentially the development of new methods.

Our study is limited primarily by the lack of granular epidemiologic data to corroborate stakeholders’ responses. Due to the lack of directly acquired clinical data, there is no confirmation of findings or adjustment for confounders. For example, there was no age standardization based on country age distribution. Despite the presence of CKD databases in some nations, population-representative surveys to estimate kidney disease prevalence are rare, especially from resource-poor regions [18]. Therefore, our study can only serve as hypothesis generating on the basis of on-the-ground observations, and as a starting point for future studies and interventions.

The data in this study, although limited, was collected from national representatives or leaders in kidney care, and their answers were based on their perspectives on practice type and location, knowledge, and expertise in kidney disease. Responses were completely internally consistent, meaning that multiple stakeholders in the country recognized the identified hotspots as such. The internal consistency in their responses provides strong rationale for investment in regional, national, and subnational data on CKD prevalence and incidence. Furthermore, the heterogeneity in associated contributors (e.g., high prevalence of diabetes among native communities versus agricultural work in the MeN hotspots) highlights the importance of targeted solutions, informed by data. In some cases, where granular data were available the survey data generally yielded responses consistent with existing literature [34], such as the contributors to the CKD hotspots in Latin America. These are often attributed in scientific literature to climate, cultural, environmental factors, and associated with residence near the agricultural industry [10, 35, 36], as was also reported in our survey.

## Conclusions

In-country stakeholders in kidney disease care identify a remarkable number of sub-national geographic regions where they perceive a disproportionately high prevalence of CKD, relative to other regions within their country. While CKD hotspots were certainly reported in known CKDu-endemic areas, this survey also identified new CKD hotspots. Regions or populations with a high prevalence of CKD should be prioritized for CKD screening, risk factor management, treatment, and research. Since unexpectedly young populations may be affected within these reported hotspots, population-representative screening would be most appropriate. Detection requires a pathway to care, so any comprehensive screening efforts will require *a priori* capacity for diagnostics and therapeutics, but also ascertainment of understudied risk factors such as occupational history and use of alternative medicines. Therefore, a collaborative effort by healthcare providers, health systems, and policy makers is imperative to protect vulnerable populations. Our preliminary findings should encourage researchers to engage in further conversations with local policymakers and conduct more structured studies on the CKD hotspots and disproportionately affected populations identified in our survey.

## Supporting information

S1 Checklist Inclusivity in global research. (DOCX)

S1 Fig Chronic kidney disease causes in Latin America and other countries. (DOCX)

S1 Table CKD hotspot descriptions from survey free-text responses. (DOCX)

S1 Data (XLSX)
