# Supplementary material for: Regional hotspots for chronic kidney disease: A multinational study from the ISN-GKHA
Source: PLOS Glob Public Health. 2024 Dec 5;4(12):e0004014. doi: 10.1371/journal.pgph.0004014 (PMC11620454; doi:10.1371/journal.pgph.0004014)
Supplement: S1 Fig — (DOCX) [file pgph.0004014.s002.docx]

**S1 Fig. Chronic kidney disease causes in Latin America and other countries.**

Abbreviation: HTN = hypertension
