# Supplementary material for: Regional hotspots for chronic kidney disease: A multinational study from the ISN-GKHA
Source: PLOS Glob Public Health. 2024 Dec 5;4(12):e0004014. doi: 10.1371/journal.pgph.0004014 (PMC11620454; doi:10.1371/journal.pgph.0004014)
Supplement: S1 Table — (DOCX) [file pgph.0004014.s003.docx]

**S1 Table. CKD hotspot descriptions from survey free-text responses.**

| **ISN Region** | **Country** | **Responses*** |
| --- | --- | --- |
| **Africa** |  |  |
|  | Burkina Faso | "In the gold-panning population" |
|  | Ghana | May have some among rural farmers working more in the sun without our knowledge |
|  |  | There is a high frequency of high risk APOL1 allele in one ethnic group compared to others. Our CKD patients are mostly young between 20 to 50 years. |
|  |  | Frequency of APOL1 high risk variants is highest among the Akan population so we suspect that there will be regional variations in the rate of kidney disease in the country, but we have not had data to support that. |
|  | Namibia | Yes, urban areas have a higher rate of risk factors for CKD |
|  | Niger | "In my center, which covers at least 4 regions, more than half the dialysis patients come from a single region" |
|  | Togo | "Northern region" |
|  | Zambia | More in urban regions versus rural based and so is hypertension |
| **Eastern and Central Europe** |  |  |
|  | Turkey | Due to regional nutrition habits, some parts of the country suffer higher risk of kidney disease |
| **Latin America** |  |  |
|  | Argentina | Poor areas have a higher risk |
|  |  | "There are areas of higher incidence of CKD and admission to dialysis in Argentina. To date, there has not been a study that adequately evaluates the causes of CKD." |
|  | Bolivia | "There may be unreported and/or unidentified cases of CKDu in regions with extreme working conditions such as sugar cane cultivation. Self-medication and lack of control over over-the-counter drug sales." |
|  | Colombia | "Special populations, agricultural areas, mining" |
|  |  | "Aboriginal and black populations of the Pacific coast have a very high prevalence of hypertension." |
|  | Costa Rica | "Chorotega region has the highest" |
|  | Ecuador | "Sea level populations with higher incidence of diabetes and hypertension" |
|  |  | People geographically [isolated] without access to medical services |
|  | El Salvador | "There are clear areas of agricultural zones with high temperatures." |
|  | Guatemala | "In agricultural communities of the south coast it is evident, and we have demonstrated that there is a population vulnerable to CKD." |
|  |  | "Young agricultural worker at risk for kidney disease of nontraditional cause" |
|  |  | "The southern coastal area and regions located less than 300 meters above sea level present with cases of Mesoamerican nephropathy" |
|  | Haiti | "Hypertensives and diabetics, the poor with no access to healthcare" |
|  | Mexico | "if we have contacted areas with a high prevalence of CKD, such as the area of Tierra Blanca, Veracruz." |
|  |  | It has been suggested that Mexico may have newly identified CKD of unknown origin (CKDu) hotspots. Studies from Tierra Blanca, a rural region in Mexico, have shown that the prevalence of probable CKD is high (25%) among the population, of which almost half of the cases had no known traditional risk factors, such as diabetes or hypertension. Lozano-Kasten et al. screened 394 children residing in a community with high incidence of CKD of unknown origin in Jalisco; he reported that 45.7% of children had persistent albuminuria. In the same community, in a cross-sectional study comparing CKD prevalence in Poncitlan residents to those residing in other Jalisco municipalities, a twofold and threefold increase in the prevalence of CKD and proteinuria, respectively, was found in adults residing in Poncitlan. In children the prevalence of CKD was similar (3.4% vs 0.7%) in the two populations; however, the prevalence of proteinuria was tenfold higher in children residing in Poncitlan in comparison to other municipalities. |
|  |  | "There are some regions considered CKD hotspots" |
|  | Panama | Center and South region of the country |
|  | Paraguay | "Agricultural workers" |
|  | Peru | "The northern population of the country suffers more from nephrolithiasis" |
|  |  | "It is suspected that there are patients with CKD as a result of environmental contamination by metals from unregulated mining" |
| **NIS and Russia** |  |  |
|  | Azerbaijan | Based on personal observations population in Azerbaijan has increased incidence of IgA Nephropathy, Nephrolithiasis, CKD, and Diabetic Nephropathy |
|  | Ukraine | Medical care in regions is different so the results for CKD are different too |
| **North America and the Caribbean** |  |  |
|  | Barbados | Patients with diabetes and hypertension |
|  | Canada | First nations populations. Rural remote populations. |
|  | St. Vincent and the Grenadines | I believe that on the Grenadine Island, there is an increased incidence of advanced kidney disease. |
|  | United States | Generally related to geographic areas with higher rates of obesity and hypertension as well as lower socioeconomic status |
|  |  | Higher in poor areas around the Mississippi delta |
| **North and East Asia** |  |  |
|  | China | Geographical difference |
|  |  | There are certain areas with relatively high incidence of kidney failure due to unknown reasons. |
|  | Japan | The rate of CKD depends on the number of nephrologists in each region. |
|  |  | In hot and dry weather region. |
|  | Taiwan | CKD rate is higher in the south regions |
| **Oceania and South East Asia** |  |  |
|  | Australia | Hot spots have been identified based on national health surveys. Tend to track with socioeconomic disadvantage and rural/remote areas. |
|  |  | There are known geographical variances in CKD prevalence. CKD Hot Spots. |
|  | New Caledonia | "High prevalence of dialysis in New Caledonia" |
|  |  | "Melanesian and Wallisian" |
|  | Philippines | Some areas in the country show higher rates of CKD but data need to be validated since population density, reporting and data collection are higher/better in urban areas. |
|  | Thailand | Culture |
|  |  | North and North-Eastern Region of Thailand have quite high prevalence of CKD due to specific risk factors (genetics, traditional medicine and renal stones) |
|  | Vanuatu | Urban & peri-urban centres - Higher risk of noncommunicable diseases and subsequent kidney failure. |
|  | Vietnam | The response mainly depends on our observations and no available data |
| **South Asia** |  |  |
|  | India | CKDu is seen in Uddanam region of Andhra Pradesh and a few pockets in Goa and Odisha. It is common among agricultural field workers. |
|  |  | Infrastructure is different in different parts |
|  |  | There are CKDu clusters |
|  | Sri Lanka | CKDu or CINAC is seen in some agricultural areas in the country. |
|  |  | Kidney failure in certain geographic regions. in these areas there is a very high risk of kidney failure, even in younger age groups. |
| **Western Europe** |  |  |
|  | Andorra | Socialized medicine. All clients come to same place for healthcare |
|  | Austria | Related to body mass index and diabetes prevalence |
|  | France | "High rate of CKD in overseas territories due to high incidence of diabetes" |
|  | Iceland | Families with ADPKD and interstitial nephritis |
|  | Norway | Probably in urban areas, especially in areas of lower socio-economic level and higher prevalence of obesity/diabetes/tobacco smoking etc. and lower health literacy level. |
|  | Portugal | Poorest and most uncultured regions and life habits. |
|  | Spain | "Higher prevalence of diabetes in the Canary Islands" |
|  |  | higher incidence of kidney replacement therapy in Mediterranean regions from Catalonia to Murcia, as well as in Canary Islands and in depopulated regions with an elderly population |
|  |  | "There are areas with a very high rate of diabetes" |
|  | Sweden | Hereditary, socio-economic |
|  | Switzerland | "Disparities for rural populations versus those living near the capital's major urban center. " |
|  | United Kingdom | There is no overall difference in the prevalence of kidney disease by public health England across high-risk groups, but there is an increased risk of progression to end stage kidney disease in specific groups, regional variation maps on to demography - e.g. increased incidence and prevalence in people of black and south Asian ethnicity. |
|  |  | Health inequalities are prevalent in England |
